# Midgut Volvulus Evolving From Internal Herniation Following Roux-en-Y Gastric Bypass Surgery

**DOI:** 10.7759/cureus.50038

**Published:** 2023-12-06

**Authors:** Faleh M Alotaibi, Mohammed A Alharbi, Bader S Alanazi, Dunya Alfaraj, Hamza Aldossary

**Affiliations:** 1 College of Medicine, Imam Abdulrahman Bin Faisal University, Dammam, SAU; 2 Emergency Medicine, Imam Abdulrahman Bin Faisal University, King Fahad University Hospital, Dammam, SAU; 3 Radiology, King Fahad University Hospital, Dammam, SAU

**Keywords:** h. pylori infection, bariatric surgery complications, roux-en-y gastric bypass (rygb), internal herniation, midgut volvulus

## Abstract

In the literature, midgut volvulus is a well-known surgical complication following gastric bypass surgery that is serious and necessitates an immediate intervention. Here, we report a case of internal herniation that was misdiagnosed twice but eventually managed appropriately. A 27-year-old male with a surgical history of Roux-en-Y gastric bypass came to the emergency department complaining of severe epigastric abdominal pain. Two months earlier, he had a similar pain which was treated with *Helicobacter pylori* eradication therapy. Despite completing the eradication therapy, the pain reoccurred. Computed tomography angiography showed a filling defect in the superior mesenteric artery that was followed by a diagnostic laparoscopy ending with internal hernia reduction. Physicians should consider internal herniation as a differential diagnosis for every patient with a history of gastric bypass surgery presenting with abdominal pain.

## Introduction

Roux-en-Y gastric bypass (RYGB) is one of the most effective bariatric surgery approaches. It provides significant weight loss and improvement of medical comorbidities with acceptable morbidity and complication rates. RYGB can be completed either retrocolically or antecolically using an open or, more commonly these days, laparoscopic method. During the procedure, the jejunal segment (Roux limb) and the gastric pouch are anastomosed after the gastric pouch is created from the proximal end of the stomach. Subsequently, a common channel is created from the jejunojejunal anastomosis to the cecum by the Roux and biliopancreatic limbs being anastomosed. However, some complications can occur following the procedure. Internal hernia is one common complication of RYGB wherein a part of the small intestine passes through a mesentery defect leading to volvulus, ischemia, perforation, and death. Midgut volvulus is a serious health event where the intestine rotates around itself, which can threaten the blood supply. Ambiguous anatomy, non-specific symptoms, and insignificant laboratory findings make the diagnosis of internal hernia a challenging process. In this article, we demonstrate an incident of small bowel obstruction attributed to midgut volvulus due to an internal hernia after two years of RYGB along with our approach to manage this condition [1,2].

## Case presentation

A 27-year-old male with a history of morbid obesity who had undergone laparoscopic RYGB two years ago presented to our emergency department with a one-day history of severe epigastric pain. He reported that the pain woke him from sleep and was not relieved by analgesia but partially relieved by leaning forward. The pain was associated with nausea but not vomiting. He denied any trauma or trigger for the pain. Two months before the presentation, he had experienced similar pain. He visited a private hospital, where he was diagnosed with a *Helicobacter pylori *infection and completed eradication therapy. Since that event, he felt well until two days ago when he experienced similar pain. He went to the same private hospital, where he was rediagnosed with *H. pylori* and discharged on hyoscine and cefuroxime. However, he did not feel any improvement, so he presented to our emergency department.

**Figure 1:**
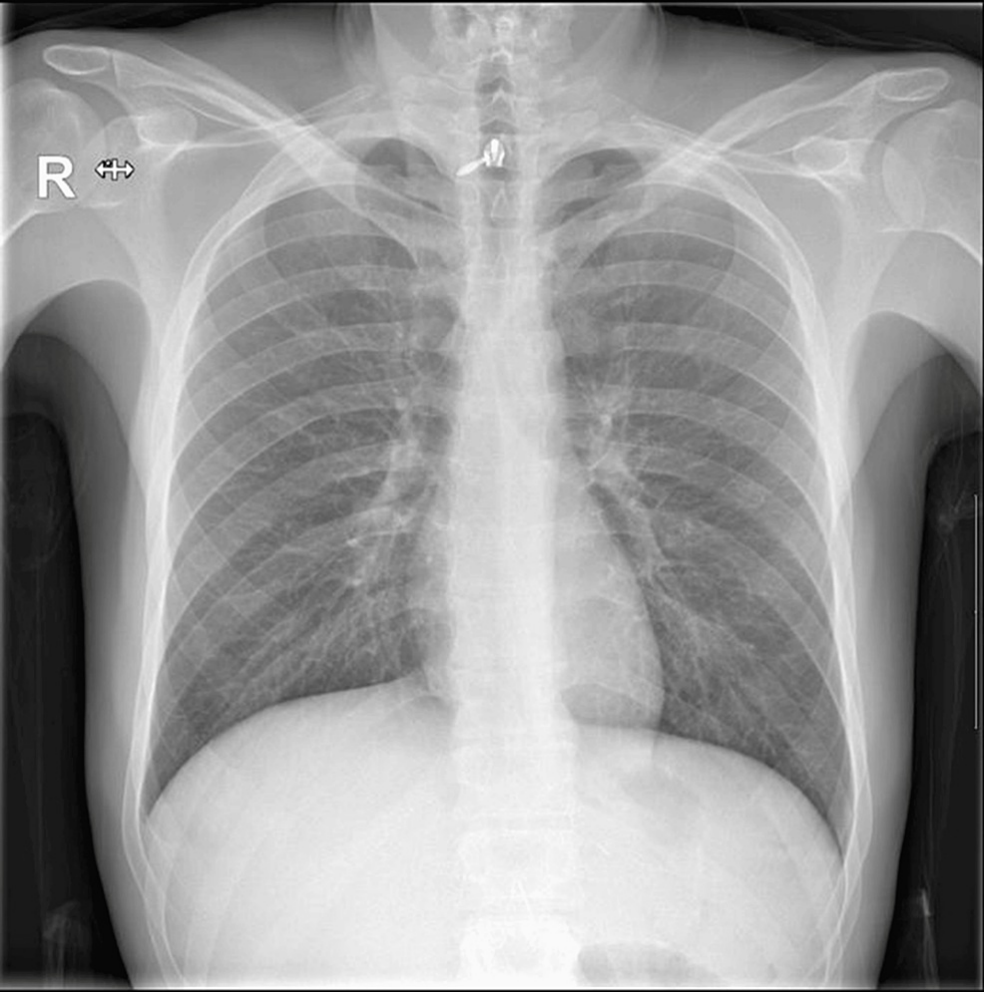
Chest X-ray showing normal findings.

**Figure 2:**
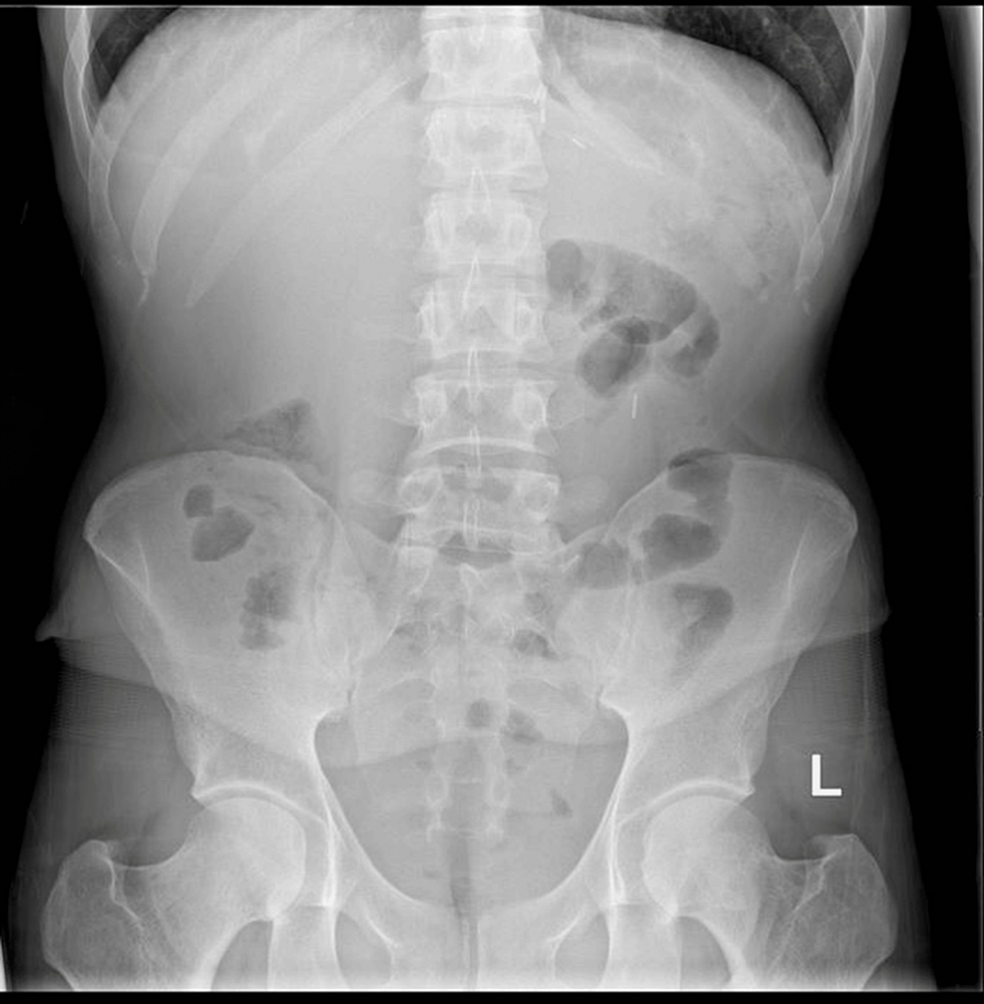
Abdominal X-ray showing normal findings.

**Figure 3:**
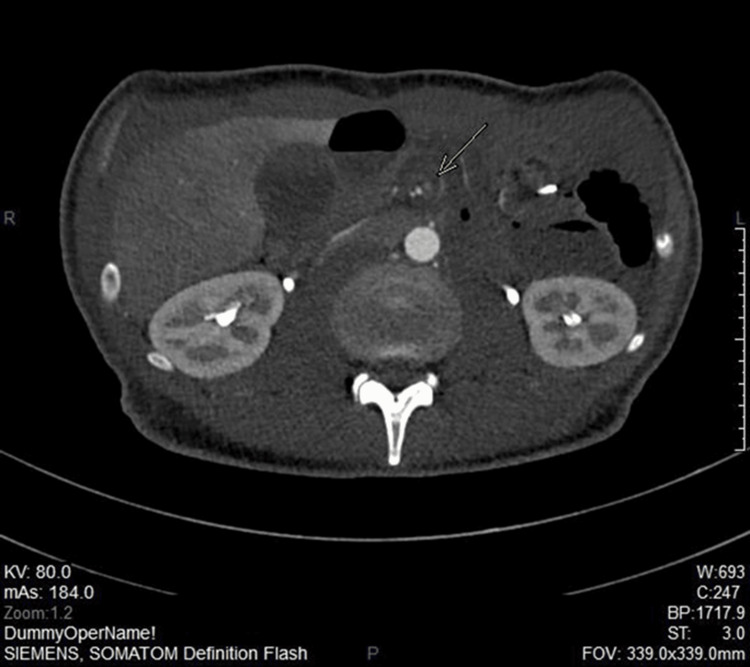
The middle portion of the superior mesenteric artery (SMA) is not opacified with contrast with the swirling of the midgut around the SMA, which is suggestive of midgut volvulus.

Upon presentation, vital signs revealed that he was afebrile, hypertensive, and had a normal pulse rate. Abdominal examination showed mild epigastric tenderness, soft and lax abdomen, negative Murphy’s sign, and rebound tenderness. Laboratory findings demonstrated normal white blood cell count, normal renal function test, and normal serum lactate level (Table 1). Chest and abdominal X-rays were unremarkable, with no pneumoperitoneum (Figures 1, 2). Therefore, a computerized tomography angiography (CTA) of the abdomen was ordered during the emergency room visit, which showed a complete filling defect in the superior mesenteric artery with good distal filling due to collateral along with swirl sign, suggestive of midgut volvulus (Figure 3). Medications given during the emergency room stay and four days of admission are illustrated along with their corresponding doses in Table 2. The patient underwent diagnostic laparoscopy converted to laparotomy with subsequent internal hernia reduction and closure of the defect.

**Table 1:** Laboratory values shown are within the normal range.

Labs	Values (reference range)
White blood cell count	10.0 k/µL (4.0–11.0)
Red blood cell count	4.46 Mil/µL (4.7–6.1)
Hemoglobin	14.6 g/dL (13.0–18.0)
Hematocrit	42.2% (42.0–52.0)
Platelet	428 k/µL (140–450)
Blood urea nitrogen	8 mg/dL (6–24)
Creatinine	0.9 mg/dL (0.8–1.3)
C-reactive protein	9.11 mg/dL (0.3–1.0)
Lactate	1.1 mmol/L (<2)

**Table 2:** Medications the patient received in the emergency room and during admission.

Medications	Dose and frequency
IV pantoprazole	40 mg - three times a day for four days
IV paracetamol	1 g - four times a day for four days
IV lornoxicam	16 mg - once during the first encounter
Morphine	5 mg - three times a day for three days
IV diazepam	5 mg - once during the first encounter
IV ciprofloxacin	400 mg - twice a day for three days
IV metronidazole	500 mg - twice a day for four days
IV ondansetron	4mg - once during the emergency room stay

## Discussion

Bariatric surgery remains the best management option for obesity and metabolic syndrome [3]. Weight reduction achieved by such surgeries is associated with a significant reduction in metabolic syndrome-related illnesses [4,5]. However, potential complications, such as cholecystolithiasis, internal herniation, and vitamin deficiencies, can arise, which are more likely to be seen in patients who underwent RYGB than those who underwent gastric sleeve procedures [6,7]. Patients can present with intermittent herniation or even necrosis preceded by strangulation. Moreover, considerable weight loss is linked with a higher likelihood of internal herniation [8]. While most cases of cecal volvulus are caused by an overly mobile cecum twisting on itself, there have been reports of internal herniation caused by Meckel’s diverticulum, incarceration within a giant ventral hernia, endometriosis, and herniation through the Foramen of Winslow. RYGB is a frequently performed bariatric procedure in the United States. An internal herniation is a conceivable sequela with a proclaimed occurrence of 1-11% in the late postoperative phase. Following a retrocolic approach, three anatomical locations for internal herniation exist, namely, between the roux limb mesentery and the transverse mesocolon, known as the Petersen’s space, straight across the transverse mesenteric defect, or through the mesenteric aperture at the back of the biliary limb [9].

Patients with bowel obstruction commonly complain of acute and occasional abdominal pain, nausea, vomiting, and loss of ability to defecate or pass flatus. A swirl sign can be shown on a CT scan in the event that the blockage is a closed-loop obstruction. This observation shows attenuation of mesenteric fat and soft tissue with neighboring segments of the intestine around the twisted intestinal arteries. According to a recent study, a sensitivity of 64% is maintained by the swirl sign and a positive predictive value of 21% in detecting a small intestine volvulus [10].

A case with symptoms of a small intestinal obstruction may have adhesions, hernia, tumor, intussusception, a small bowel hematoma, or pathology pertaining to lumen patency. In the United States and Western Europe, volvulus accounts for only 4-15% of mechanical small bowel blockages. The sigmoid, accounting for up to 80% of large intestine involvement, is the most prevalent, followed by the cecum, which accounts for up to 20% of large intestine involvement [10].

Primary or secondary small bowel volvulus can occur. Patients with a virgin abdomen and no anatomic anomalies that would predispose them to developing a volvulus have primary volvulus. This accounts for 10-22% of all volvulus instances in the Western world. In the United States, secondary volvulus is significantly more prevalent. It is seen in people with congenital or acquired abdominal diseases, such as adhesions, tension bands, or anatomic abnormalities [10]. In a similar case, a patient with internal herniation and volvulus was treated with open laparotomy to achieve adequate visualization [1].

A high index of doubt should be maintained for bowel obstruction in patients with a past surgical history of RYGB to allow for immediate diagnosis and prevent bowel resection. Unfortunately, our patient was treated for *H. pylori* solely twice in a private hospital, receiving the same diagnosis and management on the second visit even after completing the eradication regimen earlier.

## Conclusions

Internal hernia is a rare yet potentially life-threatening complication of gastric bypass surgery. Diagnosis of such a condition can be challenging, emphasizing the importance of maintaining a high index of suspicion whenever dealing with a patient with a past surgical history of gastric bypass surgery and presenting with acute or intermittent abdominal pain.
